# Stroke clinical coding education program in Australia and New Zealand

**DOI:** 10.1177/18333583231184004

**Published:** 2023-07-07

**Authors:** Monique F Kilkenny, Ailie Sanders, Catherine Burns, Lauren M Sanders, Olivia Ryan, Carla Read, Miriam Lum On, Anna Ranta, Tara Purvis, Carys Inman, Dominique A Cadilhac, Helen Carter, Stella Rowlands, Lee Nedkoff, Muideen T Olaiya, on behalf of the Australia and New Zealand Stroke Coding Working Group

**Affiliations:** 1 Monash University, Australia; 2 The Florey Institute of Neuroscience and Mental Health, Australia; 3 St Vincent’s Hospital Melbourne, Australia; 4 University of Melbourne, Australia; 5 The Victorian Agency for Health Information, Australia; 6 Australian Institute of Health and Welfare, Australia; 7 University of Otago-Wellington, New Zealand; 8 Sunshine Coast Hospital and Health Service, Australia; 9 The University of Western Australia, Australia; 10 Victor Chang Cardiac Research Institute, Australia

**Keywords:** clinical coding, education, stroke, international classification of diseases, medical records, health information management

## Abstract

**Background:**

Accurate coded diagnostic data are important for epidemiological research of stroke.

**Objective:**

To develop, implement and evaluate an online education program for improving clinical coding of stroke.

**Method:**

The Australia and New Zealand Stroke Coding Working Group co-developed an education program comprising eight modules: rationale for coding of stroke; understanding stroke; management of stroke; national coding standards; coding trees; good clinical documentation; coding practices; and scenarios. Clinical coders and health information managers participated in the 90-minute education program. Pre- and post-education surveys were administered to assess knowledge of stroke and coding, and to obtain feedback. Descriptive analyses were used for quantitative data, inductive thematic analysis for open-text responses, with all results triangulated.

**Results:**

Of 615 participants, 404 (66%) completed both pre- and post-education assessments. Respondents had improved knowledge for 9/12 questions (*p* < 0.05), including knowledge of applicable coding standards, coding of intracerebral haemorrhage and the actions to take when coding stroke (all *p* < 0.001). Majority of respondents agreed that information was pitched at an appropriate level; education materials were well organised; presenters had adequate knowledge; and that they would recommend the session to colleagues. In qualitative evaluations, the education program was beneficial for newly trained clinical coders, or as a knowledge refresher, and respondents valued clinical information from a stroke neurologist.

**Conclusion:**

Our education program was associated with increased knowledge for clinical coding of stroke. To continue to address the quality of coded stroke data through improved stroke documentation, the next stage will be to adapt the educational program for clinicians.

## Introduction

Globally, stroke is a leading cause of death, morbidity and disability, and it constitutes a considerable public health and socioeconomic burden (Feigin et al., 2022). Governments worldwide are developing surveillance systems to generate data that can be used to inform stroke prevention and management programs, to reduce the burden of stroke (Feigin et al., 2014). Routinely collected hospital administrative data are increasingly being used to monitor the incidence and outcomes of stroke (Olaiya et al., 2022), and provide complementary evidence to population-based epidemiological studies of the burden of stroke (Cadilhac et al., 2016; Thayabaranathan et al., 2022). Therefore, accurate documentation of information in hospitals and diagnostic coding are essential to improve the quality of research based on administrative data.

In Australia and New Zealand, clinical coders are responsible for data abstraction from medical records, and allocation and sequencing of diagnostic coding. The term “clinical coder” is inclusive of university-degree qualified health information managers (HIMs) and others who work as clinical coders with sub-tertiary coding education (Santos et al., 2008). In Australia and New Zealand, clinical coders use the *International Statistical Classification of Diseases and Related Health Problems*, 10th revision, Australian modification (ICD-10-AM) codes, in accordance with the Australian Coding Standards (ACS).

In previous research from this collaboration, we found some discordance in Australia between the ICD-10-AM codes for stroke recorded in hospital administrative data, and the clinician-assigned diagnoses documented in the Australian Stroke Clinical Registry (Ryan et al., 2021). In this prior study, the level of concordance ranged from 80% for transient ischaemic attack, to 72% for ischaemic stroke, and 50.8% for unspecified stroke type (Ryan et al., 2021). Discrepancies in stroke coding have also been reported in studies conducted in several other countries, including the United States, France and Italy (Baldereschi et al., 2018; Chang et al., 2016, 2019; Haesebaert et al., 2013; Jones et al., 2014).

Barriers to high-quality clinical coding include incomplete or poor clinical documentation, variability in coding training and limited opportunities for continued education of clinical coders (Doktorchik et al., 2020; Huston, 2009; Otero Varela et al., 2022; Resslar et al., 2019; Santos et al., 2008). Ongoing coding education is associated with increased specialised coding knowledge and improved coding accuracy and consistency (Lorenzoni et al., 1999; Paydar and Asadi, 2021; Santos et al., 2008). It was recommended that expert clinical staff be included in coding training to enhance clinical relevance and to facilitate communication between clinicians and clinical coders (Bramley and Reid, 2005; Santos et al., 2008). Researchers and stroke clinicians have advocated for the need for a standardised disease-specific clinical documentation, and coding education program for stroke, that targets clinicians and clinical coders (Ryan et al., 2021). However, it is unclear whether these types of education programs improve knowledge of clinical coding conventions required to accurately classify stroke. In this study, we describe the development, implementation and evaluation of a stroke-specific coding program for clinical coders in Australia and New Zealand.

## Method

### Development of the education program and evaluation surveys

An online education program was co-developed between 2019 and 2021, with input from a range of experts and stakeholders, including clinical coders, epidemiologists and stroke clinicians from the Australia and New Zealand Stroke Coding Working Group. This working group was established in 2020, by author MK, as an initiative to improve clinical documentation and coding for stroke in Australia and New Zealand. The group comprises HIMs working in hospitals, government institutions and academia; database managers; epidemiologists; neurologists; statisticians; and representatives from the Australian Stroke Clinical Registry, the Australian Institute of Health and Welfare and the New Zealand National Stroke Network. Members were recruited from the Australian Stroke Clinical Registry program, and snowballing via networks of HIMs and the broader stroke network (Supplemental Acknowledgements).

The draft online education program comprised (a) live delivery of eight structured modules based on stroke clinical coding and documentation conventions; (b) assessment of participant knowledge of stroke and clinical coding of stroke, immediately before and after the education session; and (c) evaluation of the education program after the session. The eight education modules covered the rationale for coding of stroke, understanding the brain and stroke, clinical management of stroke, ICD-10-AM coding standards, good coding and clinical documentation practices, stroke-specific coding decision trees, and scenarios provided as examples (Supplemental Box S1). Contents included in the modules were obtained from the grey literature, such as a flow chart from the New Zealand Ministry of Health on coding of stroke (New Zealand Ministry of Health, 2019).

### Knowledge assessment survey

The surveys were designed to be self-administered and completed via a purposely designed database created using the online Research Electronic Data Capture tool (Harris et al., 2009, 2019). Effectiveness of the education delivery was assessed at both Kirkpatrick Level 1 (Reaction) and Level 2 (Knowledge). The knowledge assessment survey (Supplemental Box S2) was administered immediately before and after the delivery of the education session. This survey comprised three questions on participants’ demographic information (i.e. age group, level of education, and residential postcode to determine location (Australian Bureau of Statistics, 2021)), followed by 12 close-ended, multiple-choice questions focused on the topics covered in the education session (Kirkpatrick Level 2).

### Program evaluation survey

The program evaluation survey (Supplemental Box S2) comprised 12 Likert-scale agreement questions, focused on assessing the overall quality of the content included in the education session. This included questions on the participants’ perceived level of satisfaction with each module, appropriateness of the education information, organisation of materials, extent of the presenter’s knowledge and whether they would recommend the session to colleagues. The evaluation survey also contained two open-ended questions for participants to report the most beneficial components of the live, online session, and to provide feedback about any potential areas of improvement (Kirkpatrick Level 1). To minimise any contamination of the effect of the education program, participants were encouraged to complete the pre- and post-education survey without referring to other materials.

### Review of education modules and surveys by the working group

Drafts of the education modules and surveys were reviewed by the Australia and New Zealand Stroke Coding Working Group. Members of the working group participated through online meetings, or via email, to help refine the contents of the education program.

### Pilot phase of the education program

A 60-minute pilot education program was delivered online in October 2020 to 25 third-year health information management university students. Participants were required to complete pre- and post-education session knowledge assessment surveys and the program evaluation survey. The education program was refined based on data obtained from this pilot session. These refinements included: (a) increasing the duration of the education session to 90 minutes, to allow more time for detailed presentation of the content; (b) reducing the number of knowledge assessment questions from 18 to 12; and (c) designing electronic interactive polls to facilitate participant responses to the scenarios used to demonstrate good clinical documentation practices. The clinical information and coding-related content of the modules were also refined based on additional feedback received from an experienced stroke neurologist (LMS), three final-year health information management university students, and six members of the working group who were not involved in the initial review of the program. A second pilot session was undertaken for seven clinical coders from an Australian private hospital to test changes to the program and survey, after which no further refinements were recommended prior to the commencement of implementation.

### Participant recruitment

Clinical coders working across Australia and New Zealand were invited to participate in a 90-minute online education session, via advertisements published in the March 2022 edition of the Health Information Management Association of Australia newsletter, and through clinical coder workforce mailing lists of state and federal health departments in Australia and New Zealand. Following registration, participants were emailed an explanatory statement outlining voluntary participation information 24 hours prior to their session.

### Implementation and evaluation of the education program

A total of four education sessions were conducted between April and June 2022 (Supplemental Box S3). To ensure sessions were conducted at a time appropriate for each time zone, sessions were split by geographic locations in Australia and New Zealand. Based on the presenter’s expertise and the pilot phase results, an epidemiologist/HIM (MFK) covered the rationale for coding of stroke and scenarios. A neurologist (LMS) delivered the session on understanding the brain and stroke, and clinical management of stroke. An HIM (AS) delivered standards, practices and decision trees for coding, and an epidemiologist (MO) covered clinical documentation. An iterative approach was taken, where changes to the education session were implemented for subsequent sessions based on feedback received.

### Data analysis

Descriptive statistics were used to summarise characteristics of participants and responses to the knowledge assessment survey. McNemar’s tests were used to assess participants’ change in knowledge after the education session, compared to their knowledge recorded before attending the education session. A two-sided p-value <0.05 was considered statistically significant.

All free-text data were summarised using inductive thematic analysis (AS, HIM). Triangulation, by including the collection of quantitative and qualitative data, was used to evaluate the education session, in order to improve the understanding of results and gain insights into common themes (Carter et al., 2014; Denzin, 1978). A pragmatic sample of ~25% of responses were dual coded by an independent reviewer (CI, HIM) to check interpretation against the data. Furthermore, ongoing discussions between the research team were used to ensure data were being interpreted and summarised to best reflect the intended meaning. Data were analysed using Microsoft Excel™ and STATA/MP 17.0 (StataCorp, College Station, TX, USA).

### Ethics approval and data availability

Ethics approval was obtained from the Monash University Human Research Ethics Committee in Victoria, Australia (Project ID: 30395). The data that support the findings of this study are available from the corresponding author upon reasonable request. The educational materials from the session are available online (www.auscr.com.au/stroke-coding-working-group/).

## Results

### Characteristics of participants

A total of 831 clinical coders registered for one of the four online sessions, with 615 (74%) attending (Supplemental Table S1). The majority of attendees were aged between 40 and 59 years (60%), had a bachelor or higher degree (61%), were from Australia (77%), and were located in a metropolitan area (61%; Table 1). Of participants who attended, 404 (66%) completed both the pre- and post-education session knowledge assessment surveys. There were no statistical differences between demographic characteristics of participants who completed both surveys and those who did not (Table 1).

**Table 1.** Characteristics of participants in the Australia and New Zealand stroke coding education sessions according to completion of pre- and post-education session knowledge assessment surveys.

Participant characteristics	Overall *N* = 615 *n* (%)	Completed both surveys *N* = 404 *n* (%)	Did not complete both surveys *N* = 211 *n* (%)	*p*-Value
Age group (years)				
20–29	47 (8)	29 (7)	18 (9)	0.97
30–39	131 (21)	87 (22)	44 (21)	
40–49	178 (29)	119 (29)	59 (28)	
50–59	189 (31)	123 (30)	66 (31)	
60+	70 (11)	46 (11)	24 (11)	
Level of education				
Postgraduate degree	91 (15)	57 (14)	34 (16)	0.57
Graduate diploma/certificate	49 (8)	34 (8)	15 (7)	
Bachelor degree	235 (38)	156 (39)	79 (37)	
Diploma	65 (11)	38 (9)	27 (13)	
Certificate	135 (22)	90 (22)	45 (21)	
Year 12/senior secondary certificate	25 (4)	20 (5)	5 (2)	
Year 11 or below	15 (2)	9 (2)	6 (3)	
Country/state				
Australia				
ACT/NSW	51 (8)	26 (6)	25 (12)	0.28
Northern Territory	14 (2)	9 (2)	5 (2)	
Queensland	127 (21)	83 (21)	44 (21)	
South Australia	49 (8)	37 (9)	12 (6)	
Tasmania	20 (3)	14 (3)	6 (3)	
Victoria	208 (34)	139 (34)	69 (33)	
Western Australia	7 (1)	6 (1)	1 (<1)	
New Zealand	139 (23)	90 (22)	49 (23)	
Located in a regional area^a^	241 (39)	169 (42)	72 (34)	0.06

ACT: Australian Capital Territory; NSW: New South Wales.

a Based on residential postcode (Australian Bureau of Statistics, 2021).

### Quantitative evaluation of the education session

Among respondents who completed both pre- and post-education session knowledge assessment surveys, there were improvements in knowledge of stroke and stroke coding standards (*p* < 0.05) for 9 of 12 knowledge assessment questions post-education session, compared to pre-education session (Table 2). Respondents’ knowledge did not improve significantly post-education session for questions on clinical treatments for subarachnoid haemorrhagic and ischaemic stroke, or for questions focused on improving the accuracy of stroke coding using imaging. The median number of correct answers per respondent increased from 8 of 12 questions pre-education session to 9 of 12 questions post-education session (Supplemental Table S1).

**Table 2.** Knowledge of stroke coding pre- and post-education session among 404 participants who completed both knowledge assessment surveys.

Individual questions	Pre-education session assessment *n* (%)	Post-education session assessment *n* (%)	*p*-Value^a^
1. Ischaemic stroke definition	355 (88)	372 (92)	0.01
2. Stroke is a form of cardiovascular disease	305 (76)	370 (92)	<0.001
3. Types of stroke	366 (91)	384 (95)	0.003
4. Treatments for ischaemic stroke	320 (79)	335 (83)	0.10
5. Treatments for haemorrhagic stroke	102 (25)	190 (47)	<0.001
6. Clipping and coiling of aneurysm for the management of subarachnoid haemorrhage	258 (64)	264 (65)	0.55
7. Australian Coding Standard 0002 – Additional Diagnoses	314 (78)	360 (89)	<0.001
8. Other Australian Coding Standards relevant for stroke	197 (49)	266 (66)	<0.001
9. Adding specificity to intracerebral haemorrhage coding	168 (42)	242 (60)	<0.001
10. Adding specificity to ischaemic stroke coding	121 (30)	157 (39)	<0.001
11. Actions to take with non-specific clinical documentation when coding stroke	269 (67)	338 (84)	<0.001
12. Using CT results to reduce assignment of I64	391 (97)	396 (98)	0.20

CT: computerised tomography.

a Obtained from McNemar’s test.

**Figure 1.**
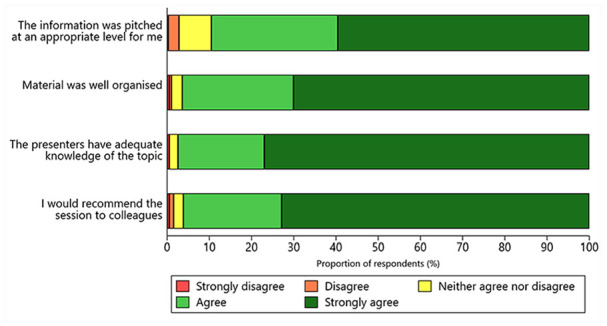
Reported satisfaction with the Australia and New Zealand Stroke Coding Education Program.

Improvements in knowledge were greater among respondents with a certificate, bachelor, or postgraduate degree education compared to those with Year 12 or below education (all *p* < 0.001; Supplemental Table S2). Overall, satisfaction with the education modules was rated as “good” or “great” (>80%; Supplemental Figure S1). Similarly, >85% of respondents reported that the contents of the modules were pitched at an appropriate level, the education materials were well organised, the presenters had adequate topic-area knowledge, and that they would recommend the education program to colleagues (Figure 1).

### Qualitative evaluation of the education session

In free-text boxes of the evaluation survey, 346 (56%) respondents provided feedback about the most beneficial aspects of the education program, and 110 (18%) suggested improvements. The feedback was categorised into themes: information provided within the session, importance/use of coded data, coded data quality, presentation and overall experience (Table 3; Supplemental Table S3).

**Table 3.** Main themes and subthemes to emerge from the thematic analysis.

Themes	Subthemes – beneficial	Subthemes – improvements
Information provided within the session	Cerebral anatomy and pathophysiology of stroke and treatment – clinician input	More information on procedures and treatments for stroke
	Use/review of ACS and coding rules, DRGs	Manifestations of stroke/deficits meeting coding criteria
	Flowcharts/coding pathway/tree helpful for coding decision-making	More complex clinical information (e.g. tandem lesion, haemorrhagic transformation, lacunar infarct)
	Inclusion of practical scenarios and polls in the session. Enjoyed the interactive component	More examples/scenarios (more complexity, including imaging, ethical documentation queries, sequelae, including additional diagnoses and procedures)
		Using flowcharts/coding pathway for assigning ICD-10-AM stroke codes with examples, showing coding pathways from books
		Documentation improvement – practical tips
		NZ/AUS specific differences (i.e. terminology, funding, statistics)
Importance/use of coded data	Use in research/registries – rationale	More information about AuSCR, ICD-10-AM codes and outcomes
Coded data quality – specificity of coding	Role of clinical coders in accurate coding Avoiding I64 When to send documentation queries Where and what (e.g. abbreviations) to look for – including imaging reports	
	Importance of accurate/complete clinical documentation	
Presentation	Delivery by specialists in the field	Inappropriate timing, too long, too rushed
	Clear explanation/logical/well presented, clear slides	Delivery of information
	Opportunity to ask questions	Slides – some slides too much information
	Pre- and post-survey to test knowledge	Answer questions during the session/sharing of Q&As, resource availability
	Appropriate length of time of session	Availability of session – more sessions
	Accessibility – online format	Accessibility issues with Zoom
	Slides/presentation/questions available for reference	Scenario discussion or layout (e.g. survey covered scenario on screen)
Overall experience	Informative/knowledge reinforcement versus refresher	Content pitched at a level more appropriate for newer clinical coders, and/or clinicians
	Experience of the clinical coder – beneficial for newer clinical coders, regional areas	
	Clarity on stroke coding	
	Entirety of session	
	Education opportunity	

ACS: Australian Coding Standards; AUS: Australia; AuSCR: Australian Stroke Clinical Registry; DRG: diagnosis-related group; ICD-10-AM: *International Statistical Classification of Diseases and Related Health Problems*, 10th revision, Australian modification; NZ: New Zealand; Q&As: questions and answer.

Several comments related to the perceived difficulty-level of the content provided. For example, it was felt that the program was considered to be most beneficial for newly trained clinical coders: “I am still training, and this is extremely helpful as it can be a bit confusing sometimes,” “[the session] was great for less experienced [clinical] coders.” However, the program was also reported by several respondents to be beneficial for more experienced clinical coders to refresh their knowledge: “It was a fantastic refresher. It gave confidence to my current coding practices” and “as a [clinical] coder I am grateful for any education” and “we no longer have education sessions, so this was brilliant.” A common highlight for many respondents was the inclusion of a neurologist in the education session to present clinical information about stroke, “[it was] very good having a neurologist explain the clinical side.” A common subtheme among respondents was accessibility of the education program. Respondents enjoyed “being able to ask questions and . . . to attend via [online]” and that “this was very accessible by having it [online].”

The majority of suggested improvements for the first session of the education program related to slowing down the presentation pace to allow adequate time for completion of the pre- and post-education session knowledge assessment surveys: “there was a lot of content to take in at a fast rate!” An iterative approach was taken and the feedback was immediately acted upon following the first session when this comment was received, and similar comments rarely appeared for the remaining sessions. The most common subtheme for suggested improvements from all four sessions related to the complexity and scope of information provided within the session. Respondents suggested more advanced scenarios and increased detail about documentation queries, treatments, procedure codes, complications and imaging reports: “It might be good to have some examples of CT/MRI results to show where in the report the information can be found if it isn’t clear in the conclusion.”

Likert-scale responses to the program evaluation survey were comparable with the free-text responses. For example, approximately 84% of respondents rated the “good clinical documentation” module as “good” or “great” (Supplemental Figure S1). This aligned with the free-text comments, where respondents commonly identified the need for more practical advice including examples of clinical documentation and documentation queries. The survey statement: “The information was pitched at an appropriate level for me” had the lowest satisfaction score of the four overall Likert-scale satisfaction questions with approximately 90% of respondents rating the education program as either “good” or “great” (Figure 1). This result was supported by feedback in free-text comments that suggested the education program was useful for beginner clinical coders or as a knowledge refresher, but may not have provided new knowledge at an advanced complexity level appropriate for experienced clinical coders.

## Discussion

The stroke-specific clinical coding education program was evaluated as being beneficial for clinical coders working in the field, both in terms of knowledge improvement and participant satisfaction. Paydar and Asadi (2021) reported similar findings for an in-service workshop on coding for pregnancy, childbirth and the puerperium conducted in Iran. In that study, participants were generally satisfied with the course; found the content appropriate and agreed they would recommend the course to colleagues. Knowledge improved significantly between the pre- and post-education session assessments demonstrating the value of training courses for clinical coders (Paydar and Asadi, 2021). Comparable results were found after a same-day procedure coding training course for Australian clinical coders and following a trial ICD-11 training program in Canada (Craig 1995; Eastwood et al., 2021).

Results of the study by Ryan et al. (2021) support the need for a standardised stroke-specific clinical documentation and coding education program to improve the quality of coded data. Others linked clinical coder productivity with hours of staff training during the transition from ICD-9 to ICD-10 in the United States (Stanfill et al., 2014). While results from this previously conducted study were not statistically significant, clinical coders with more hours of training demonstrated relatively greater productivity when new concepts were introduced, indicating a return on investment for staff training time (Stanfill et al., 2014).

In Australia, clinical coders do not have mandatory requirements for ongoing professional education after they complete their initial qualification. Education, training and resource support are necessary to achieve good coding quality (Santos et al., 2008), with coding education associated with a greater level of data completeness than other factors, such as episode of care complexity or level of coding experience (Dyers et al., 2017). Clinical coders are expected to keep up-to-date with changes in medical terminology, treatments and procedures, but ongoing coding education often varies between workplaces and providers. Several respondents from this survey stated that they appreciated this professional development opportunity. This program may address a gap for disease-specific education tailored for clinical coders. According to respondents, our program was particularly beneficial for newly qualified or trainee clinical coders. Therefore, it may be important to make these education sessions available online to enable access to as many clinical coders as possible.

### Strengths and limitations

A strength of this study was the number of clinical coders who attended the education sessions, indicating the considerable interest in, and the reach of, the education program. While the number of clinical coders in Australia cannot be quantified, approximately 66% (139 of ~210) of clinical coders in New Zealand attended an education session (personal communication, Te Whatu Ora – Health New Zealand, 9 February 2023). Limitations of this study included a lack of follow-up to assess long-term retention of knowledge or implementation into practice. In addition, information on “years of coding experience” was not collected. The level of experience may have affected baseline knowledge assessment survey results, and suggested improvements to the education program could not be analysed in the context of the participant’s varied level of experience.

### Implications

Validation of stroke coding will continue to be monitored using data from the Australian Stroke Clinical Registry and New Zealand Ministry of Health, which may indicate long-term changes in coding practices. Questions with the lowest correct responses post-education were related to treatments for intracerebral and subarachnoid haemorrhagic strokes, and questions about improving the accuracy of intracerebral haemorrhage and ischaemic stroke coding. This highlights the need for more information on treatment, and where to obtain further information on specificity from the medical record (such as location of haemorrhage or thrombus/embolism). These results also suggest the wording of these questions may have been confusing for respondents, and this will be reviewed for future education sessions.

Two modules of this education program were delivered by an experienced stroke neurologist, which is a highly valued component of coding training (Bramley and Reid, 2005; Eastwood et al., 2021). As Eastwood et al. (2021) reported, participants find PowerPoint slides the least useful resource for coding education. Although, an online format may differ from face-to-face education, the pedagogical principles remain the same. To facilitate dynamic engagement with the content across varied learning styles, future iterations of this course may include more opportunities for discussion with subject matter experts and peer-to-peer learning to maximise the learning experience.

Two common subthemes for improvements to the program included further examples and specificity around the use of imaging reports and clinical documentation. Interestingly, knowledge assessment survey results indicated that clinical coders knew they could use imaging reports to improve coding accuracy, but in practice the clinical documentation linking the diagnosis and imaging report may be inadequate. Several respondents identified that they would prefer further examples around the use of imaging reports for code assignment. This is an area that will be explored in future research to determine the accuracy of the clinical diagnosis and ICD-10-AM administrative data, compared to the diagnosis obtained from written imaging reports.

Educating clinical coders about stroke is only one component of improving the quality of coded data; it is also essential to educate clinicians about the need for clear, accurate and consistent clinical documentation. If clinical documentation is absent, ambiguous or does not clearly link conditions and treatments, then the clinical coder cannot assign codes according to the ACS (Australian Consortium for Classification Development, 2022). Clinical documentation and discharge summaries are typically completed by the most junior members of the clinical team (e.g. interns and residents). Our team is planning education sessions for release in 2023, aimed at increasing clinicians’ understanding of clinical coding and improving the quality of clinical documentation of stroke.

## Conclusion

This Stroke Coding Education Program, designed for Australia and New Zealand, provided an opportunity for clinical coders to receive free and easily accessible disease-specific ICD-10-AM coding education. Ongoing education for clinical coders is recommended to achieve high-quality coding, and in turn, accurate data. This novel education program increased knowledge essential for improving the quality of clinical coding for stroke in hospitals, and it will be made available to the wider clinical coding community. The modules are now being adapted for use in an educational program to improve stroke clinical documentation by clinicians.

## Supplemental Material

sj-docx-1-him-10.1177_18333583231184004 – Supplemental material for Stroke clinical coding education program in Australia and New Zealand Supplemental material, sj-docx-1-him-10.1177_18333583231184004 for Stroke clinical coding education program in Australia and New Zealand by Monique F Kilkenny, Ailie Sanders, Catherine Burns, Lauren M Sanders, Olivia Ryan, Carla Read, Miriam Lum On, Anna Ranta, Tara Purvis, Carys Inman, Dominique A Cadilhac, Helen Carter, Stella Rowlands, Lee Nedkoff and Muideen T Olaiya in Health Information Management Journal
