# Supplementary material for: Stroke clinical coding education program in Australia and New Zealand
Source: Health Inf Manag. 2023 Jul 7;54(1):25–33. doi: 10.1177/18333583231184004 (PMC11707964; doi:10.1177/18333583231184004)
Supplement: sj-docx-1-him-10.1177_18333583231184004 – Supplemental material for Stroke clinical coding education program in Australia and New Zealand [file sj-docx-1-him-10.1177_18333583231184004.docx]

SUPPLEMENTARY MATERIAL

Development, Implementation and Evaluation of the Australia and New Zealand Stroke Coding Education Program

Kilkenny MF, Sanders A, Burns C, Sanders LM, Ryan O, Read C, Lum On M, Ranta A, Purvis T, Inman C, Cadilhac DA, Carter H, Rowlands S, Nedkoff L, Olaiya MT

on behalf of The Australia and New Zealand Stroke Coding Working Group

Supplemental Tables

Box S1: Learning modules covered in education session

Box S2: Surveys utilised for pre- and post-education session

Box S3: Duration of the education sessions and flow of participants

Table S1: Change in the median number (interquartile range) of correct responses to the stroke coding knowledge assessment survey after the Australia and New Zealand Stroke Coding Education Session, overall and by session

Table S2: Example of respondent comments – beneficial aspects

Table S3: Example of respondent comments – suggested areas of improvement

Supplemental Figure

Figure S1: Reported satisfaction with each module of the Australia and New Zealand Stroke Coding Education Program

Supplemental Acknowledgements

Co-investigators from the Australia and New Zealand Stroke Working Group

Supplemental Box S1: Learning modules covered in education session

| **Topic** | **Learning outcomes** |
| --- | --- |
| 1. Rationale for coding of stroke | To understand the importance of coding of stroke |
| 2. Understanding the brain and stroke | To understand the types and signs of stroke, and differentiate stroke from other cardiovascular diseases |
| 3. Treatments available for stroke | To learn the treatment and care options available for the different types of stroke |
| 4. Good clinical documentation | To understand how documentation impacts the quality and specificity of coding in administrative data |
| 5. ACS and coding resources for stroke | To understand how to apply general and stroke-specific ACS while coding stroke episodes |
| 6. Good coding practices | To learn coding tips and develop good coding skills |
| 7. Coding trees | To learn how to add specificity to stroke coding by understanding coding pathways for each stroke type |
| 8. Scenarios | To evaluate participants’ understanding and translation of knowledge acquired from the session using real-world coding examples |

ACS: Australian Coding Standards

**Supplementa**l **Box S2: Surveys utilised for pre- and post-education session**

| **Demographic Questions (Pre-education session)** | | | | |
| --- | --- | --- | --- | --- |
| **#** | **Question** | **Possible Responses** | **Field Attributes** | |
| 1 | Age group (years): | 1. 20-29 2. 30-39 3. 40-49 4. 50-59 5. 60+ | Radio, required | |
| 2 | Highest level of completed education | 1. Postgraduate degree level 2. Graduate diploma and graduate certificate level 3. Bachelor degree level 4. Advanced diploma and diploma level 5. Certificate level 6. Year 12 or Senior Secondary Certificate of Education 7. Year 11 or below | Radio, required | |
| 3 | Postcode (residential) |  | Text, required | |
| **Knowledge Assessment Survey (Pre- and post-education session)** | | | | |
| **#** | **Question** | **Possible Responses** | **Field Attributes** | |
| 1 | Which of these describes an ischaemic stroke? | 1. Rupture of cerebral vessel 2. Occlusion of a cerebral vessel 3. Aneurysm of a cerebral vessel 4. Temporary episode of neurological dysfunction | Radio, required | |
| 2 | Is stroke a form of cardiovascular disease (ICD-10-AM stroke is classified as a cardiovascular disease)? | 1. True 2. False | Radio, required | |
| 3 | What are the main categories/classifications of stroke? | 1. Ischaemic and Subarachnoid 2. Haemorrhagic and Transient Ischaemic Attack (TIA) 3. Haemorrhagic and Ischaemic 4. Transient Ischaemic Attack (TIA) and Subarachnoid | Radio, required | |
| 4 | Which of the following procedures is/are used to manage ischaemic stroke? | 1. Cerebral angiography 2. Surgical evacuation of Haematoma 3. Fresh frozen plasma 4. Intravenous thrombolysis | Radio, required | |
| 5 | Which of the following procedures is/are used to manage haemorrhagic stroke? Please select all that apply | 1. Endovascular clot retrieval 2. Ventricular drainage 3. Surgical evacuation of Haematoma 4. Tpa infusion | Checkbox, required | |
| 6 | The clipping and coiling of an aneurysm is used to manage: | 1. Subarachnoid haemorrhage 2. Cerebrovascular disease 3. Ischaemic stroke 4. Transient Ischaemic Attack (TIA) | Radio, required | |
| 7 | Deficits of a ‘current’ stroke must meet ACS0002 to be assigned. | 1. True 2. False | Radio, required | |
| 8 | Which ACS standards do you use when coding stroke? | 1. ACS 0604, ACS 0605 2. ACS 0001, ACS 0002, ACS 0008, ACS 0010, ACS 0048, ACS 0604, ACS 0605, ACS 0016, ACS 0032 3. ACS 0016, ACS 0032, ACS 0604, ACS 0605 4. ACS 0001, ACS 0002, ACS 0008, ACS 0010, ACS 0048, ACS 0604, ACS 0605 | Radio, required | |
| 9 | What would you look for to further specify an intracerebral haemorrhage? | 1. Location 2. Artery or type of blockage 3. Cause 4. CT evidence of bleed | Radio, required | |
| 10 | What should you look for to further specify an ischaemic stroke? | 1. Brain location 2. Extent of brain damage 3. Type of blockage and type of artery 4. All of the above | Radio, required | |
| 11 | If stroke or cerebrovascular accident is documented as the final diagnosis on the discharge summary. The following actions can be taken:  (A) Code I64 as principal/additional diagnosis;  (B) Send a query to the clinician if no further specifying documentation;  (C) Consult another experienced coder; and  (D) Check documentation for treatment type and imaging results.  In what order of priority should these actions be taken? | 1. (A), (D), (B), (C) 2. (A), (B), (D), (C) 3. (C), (A), (D), (B) 4. (D), (C), (B), (A) | Radio, required | |
| 12 | To further specify a stroke at the 3rd digit level, what documentation/result should a coder look for in the medical record? | 1. OT 2. ECG 3. CT 4. White blood cell count | Radio, required | |
| **Evaluation Survey (Post-education session)** | | | | |
| *Module Feedback (please select appropriate response)* | | | | |
| **#** | **Question** | **Possible Responses** | **Field Attributes** | |
| 1 | Rationale for coding of stroke | 1. Grr 2. Ho Hum 3. Okay 4. Good 5. Great | Radio (matrix), required | |
| 2 | Understanding of brain and stroke |  |  |  |
| 3 | Treatments available for stroke |  |  |  |
| 4 | ACS standards and coding resources for stroke |  |  |  |
| 5 | Good coding practices |  |  |  |
| 6 | Coding trees (index branching) |  |  |  |
| 7 | Good clinical documentation |  |  |  |
| 8 | Scenarios |  |  |  |
| *Content (please select appropriate response)* | | | | |
| **#** | **Question** | **Possible Responses** | **Field Attributes** | |
| 9 | The information was pitched at an appropriate level for me | 1. Strongly disagree 1 2. 2 3. 3 4. 4 5. Strongly agree 5 | Radio (matrix), required | |
| 10 | The material was well organised |  |  |  |
| 11 | The presenters have adequate knowledge of the topic |  |  |  |
| 12 | I would recommend the session to colleagues |  |  |  |
| *Comments* | | | |  |
| **#** | **Question** | **Possible Responses** | **Field Attributes** |  |
| 13 | What were the most beneficial aspects of the session? |  | Notes, required |  |
| 14 | What did we miss? |  | Notes, required |  |

**Supplementa**l **Box S3:** **Duration of the education sessions and flow of participants**

|  | **Duration of session (minutes)** | **Session registrations**  **n (%)** | **Session attendees**  **n (%)** | **Completed both pre- and post-education session surveys**  **n (%)** |
| --- | --- | --- | --- | --- |
| Overall |  | 831 | 615 | 404 |
| Session 1 | 70 | 333 (40) | 280 (46) | 163 (40) |
| Session 2 | 89 | 92 (11) | 70 (11) | 47 (12) |
| Session 3 | 90 | 145 (17) | 139 (23) | 90 (22) |
| Session 4 | 90 | 261 (31) | 171 (28) | 104 (26) |
| Session 1 comprised participants from Victoria and Tasmania, Session 2 comprised participants from Western Australia, South Australia, and Northern Territory, Session 3 comprised participants from New Zealand, Session 4 comprised participants from New South Wales, Australian Capital Territory and Queensland. | | | | |

**Supplementa**l **Table S1:** **Change in the median number (interquartile range) of correct responses to the stroke coding knowledge assessment survey after the Australia and New Zealand Stroke Coding Education Session, overall and by session**

|  | **N** | **Pre-education session** | **Post-education session** | **p-Value** |
| --- | --- | --- | --- | --- |
| Overall | 404 | 8 (7, 9) | 9 (8, 10) | <0.001 |
| Level of education |  |  |  |  |
| Postgraduate Degree | 57 | 8 (7, 10) | 10 (8, 11) | <0.001 |
| Graduate diploma/certificate | 34 | 8 (7, 9) | 9.5 (8, 10) | 0.004 |
| Bachelor Degree | 156 | 8 (7, 9) | 9 (8, 11) | <0.001 |
| Diploma | 38 | 8 (7, 9) | 9 (8, 10) | 0.002 |
| Certificate | 90 | 8 (7, 9) | 9 (8, 10) | <0.001 |
| Year 12 or Senior Secondary Certificate | 20 | 8.5 (7.5, 9) | 9 (8.5, 10) | 0.002 |
| Year 11 or below | 9 | 6 (5, 8) | 8 (8, 9) | 0.018 |
| Country/State |  |  |  |  |
| Australia |  |  |  |  |
| Australian Capital Territory/New South Wales | 26 | 8 (7, 9) | 9 (8, 10) | 0.009 |
| Northern Territory | 9 | 7 (6, 8) | 7 (6, 8) | 0.37 |
| Queensland | 83 | 8 (7, 9) | 9 (8, 10) | <0.001 |
| South Australia | 37 | 8 (7, 9) | 9 (7, 10) | <0.001 |
| Tasmania | 14 | 6.5 (5, 9) | 8.5 (8, 10) | 0.010 |
| Victoria | 139 | 8 (7, 9) | 9 (8, 11) | <0.001 |
| Western Australia | 6 | 8 (7, 10) | 10 (10, 11) | 0.05 |
| New Zealand | 90 | 8 (7, 9) | 9 (8, 11) | <0.001 |
| Residential location* |  |  |  |  |
| Metropolitan | 235 | 8 (7, 9) | 9 (8, 11) | <0.001 |
| Regional | 169 | 8 (7, 9) | 9 (8, 10) | <0.001 |

*Based on residential postcode (Australian Bureau of Statistics, 2021)

**Supplementa**l **Table S2: Example of respondent comments – beneficial aspects**

| **Theme** | **Subtheme** | **Comments – beneficial aspects** |
| --- | --- | --- |
| Overall experience | Experience of the coder – beneficial for newer coders, regional areas | “I am still training, and this is extremely helpful as it can be a bit confusing sometimes.” |
|  |  | “Was great for less experienced coders” |
|  |  | “I am a beginner coder, so everything was beneficial!” |
|  |  | “In a rural setting, we only deal with rehab, so the entire course has been amazing.” |
|  | Informative / knowledge reinforcement vs refresher | “Stroke education recap - it's not something I have to code often so it's good to be re-educated on types etc” |
|  |  | “More clarity on [stroke] coding.” |
|  |  | “All informative even if to reaffirm existing knowledge.” |
| Importance/use of coded data | Use in research/registries – rationale | “Great education and a reminder that coding is more than hospital funding” |
|  |  | “Acknowledgment of the importance of correct clinical coding!” |
|  |  | “Discussion on stroke registry.” |
| Information provided within the session. | Cerebral anatomy and pathophysiology of stroke and treatment – clinician input | “Explanation on different types of stokes and aetiologies” |
|  |  | “The presentation diagnostic tests and clinical presentation” |
|  |  | “Knowledge of types of stroke and treatments for them.” |
|  |  | “Explanation of imaging types and the specificity they provide.” |
|  |  | “I found the discussion about the different types of infarction/haemorrhage and the causes very informative.” |
|  | Flowcharts/coding pathway/tree for coding decision making | “Coding trees were very helpful visually” |
|  |  | “The stroke flow diagram to assist in following the correct pathways.” |
|  | Practical scenarios and polls, interactive experience | “The scenarios at the end were helpful to cement what we just learned.” |
|  |  | “I found the entire session beneficial, but I particularly liked that I got to participate for the scenarios at the end.” |

Supplemental Table S3: Example of respondent comments – suggested areas of improvement

| **Theme** | **Subtheme** | **Comments – suggested areas for improvement** |
| --- | --- | --- |
| Information provided within the session. | More examples/scenarios | “A couple more scenarios around thromboembolic infarction would have been the icing on the cake.” |
|  |  | “A little more complex scenarios would have helped.” |
|  |  | “It would be good to see real life scenarios ([e.g.] discharge summary with imaging results).” |
|  |  | “Sequalae scenarios.” |
|  | More complex clinical information (e.g. tandem lesion, haemorrhagic transformation, lacunar) | “Tandem occlusion, haemorrhaging transformation.” |
|  |  | “More clinical information on types of stroke and underlying causes, and explanation of common terminology used by clinicians.” |
|  |  | “Explaining the different manifestations of cerebral infarctions and when they would be coded.” |
|  | Flowcharts/coding pathway | “I would have liked to see more of the actual coding pathway/index for stroke coding.” |
|  |  | “Flow-charts for easy understanding.” |
|  | Documentation improvement – practical tips | “Documentation improvement could have been more expansive for Stroke types, Stroke related conditions, deficits, underlying causes.” |
|  |  | “As a [clinical documentation specialist] I work with the clinicians and their documentation, maybe some more examples of this.” |
|  |  | “A solution to bad documentation. As a smallish regional hospital where most [strokes] are up transferred to a metropolitan hospital we usually have basic documentation and getting [medical officer] clarifications is not always an option.” |
| Presentation | Answer questions during the session / sharing of Q&As, resource availability | “I'd love a handout to help decipher between cerebral and precerebral arteries” |
|  |  | “Sending out a copy of the talk for reference.” |
|  |  | “In polling section, question/answer box blocked view of scenario.” |
|  | Timing – too long  Timing – rushed | “A little too fast in a couple of the sections, especially for less experienced coders.” |
|  |  | “Polls were good but took too long.” |
|  | Scenario discussion or layout (e.g. survey covered scenario on screen) | “When the survey came on screen, the scenario was covered so difficult to review for selecting correct answer.” |
|  |  | “The poll popped up over description/question. Not sure if closing poll would allow me back into answer.” |


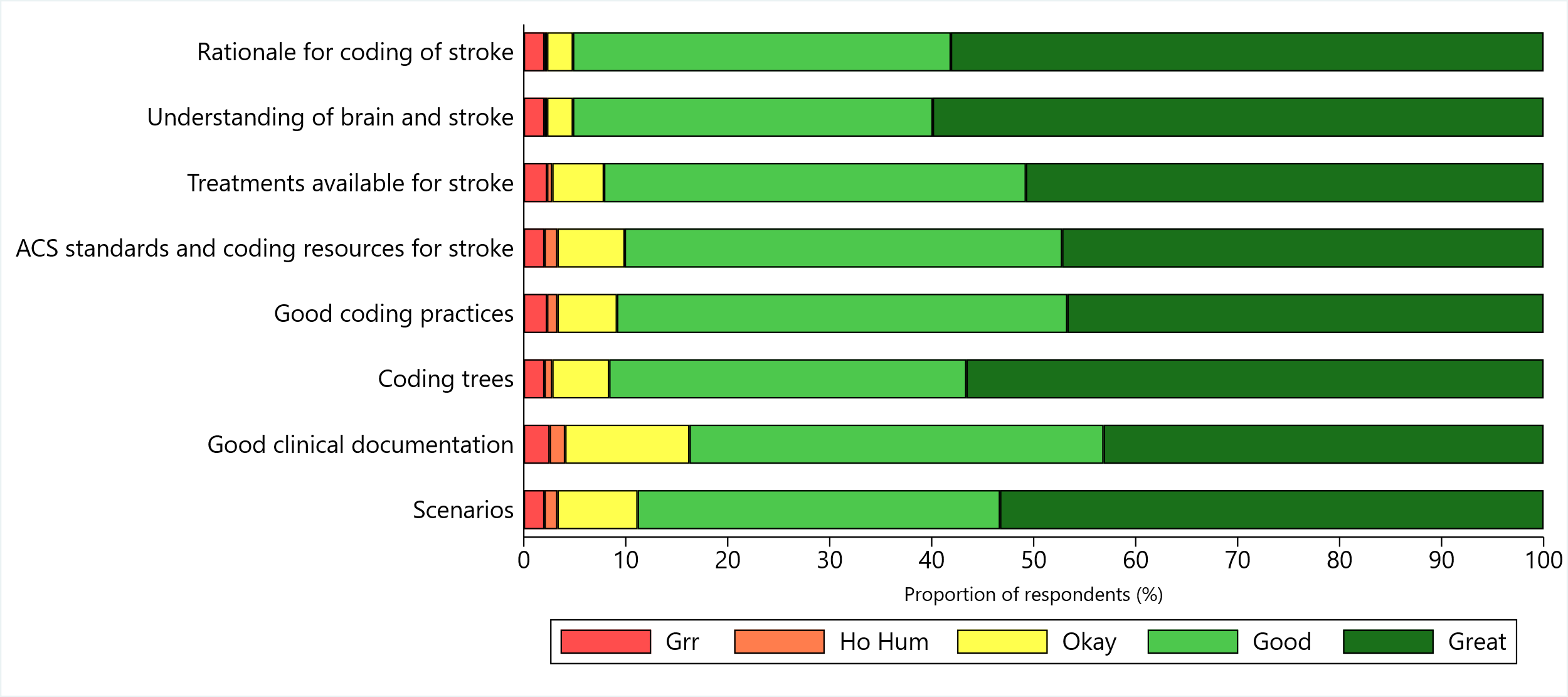


**Supplementa**l **Figure S1: Reported satisfaction with each module of the Australia and New Zealand Stroke Coding Education Program**

Supplemental Acknowledgements

Co-investigators from the Australia and New Zealand Stroke Working Group

| Member name | Affiliation |
| --- | --- |
| A/Prof Monique Kilkenny (Chairperson) | Monash University  The Florey Institute of Neuroscience and Mental Health |
| Prof Dominique Cadilhac | Monash University  The Florey Institute of Neuroscience and Mental Health |
| Ailie Sanders | Monash University |
| Helen Carter | The Florey Institute of Neuroscience and Mental Health |
| Merilyn Riley | La Trobe University |
| Dr Muideen Olaiya | Monash University |
| Dr Stella Rowlands | Sunshine Coast Hospital and Health Service |
| Carla Read | Victorian Agency for Health Information |
| Nicola Hall | Logan Hospital |
| Linda Norrie | Townsville Hospital |
| A/Prof Lauren Sanders | St Vincent’s Hospital Melbourne  University of Melbourne |
| Dr Lee Nedkoff | University of Western Australia |
| A/Prof Seana Gall | University of Tasmania |
| Miriam Lum On | Australian Institute of Health and Welfare |
| Sally Richardson | La Trobe University |
| Ngoc Dang | St Vincent’s Hospital Melbourne |
| Prof Valery Feigin | National Institute for Stroke and Applied Neurosciences |
| A/Prof Rita Krishnamurthi | Auckland University of Technology |
| Prof Anna Ranta | University of Otago Wellington |
| Dr Alan Davis | Te Whatu Ora Te Tai Tokerau |
| Jennie Carson | University of Western Australia |
| Jacquelyn Ellem | Healthscope |
| Mary Kouvas | Northern Health |
| Lara Finlayson | Clinical Coding Services |
| Justan Banihashemi | Melbourne Health |
| Susan Doyle | Monash Health |
